# Consumer Understanding and Culinary Use of Legumes in Australia

**DOI:** 10.3390/nu11071575

**Published:** 2019-07-12

**Authors:** Natalie Figueira, Felicity Curtain, Eleanor Beck, Sara Grafenauer

**Affiliations:** 1 School of Medicine, University of Wollongong, Northfields Avenue, Wollongong 2522, Australia; 2 Grains & Legumes Nutrition Council, Mount Street, North Sydney 2060, Australia; 3 Illawarra Health & Medical Research Institute, Northfields Avenue, Wollongong 2522, Australia

**Keywords:** legume, bean, dietary guidelines, vegan, vegetarian, protein, dietary fibre, culinary use, food classification, consumption

## Abstract

While health benefits of legume consumption are well documented, intake is well below recommendations in many Western cultures, and little is known regarding culinary use and consumer understanding of these foods. This study aimed to investigate consumption, knowledge, attitudes, and culinary use of legumes in a convenience sample of Australians. An online computer-based survey was used to gather data and demographic characteristics. Respondents (505 individuals answered in full or in part) were regular consumers of legumes (177/376 consumed legumes 2–4 times weekly). Chickpeas, green peas, and kidney beans were most often consumed, and were made into most commonly Mexican, then Indian and Middle Eastern meals. Consumers correctly identified protein and dietary fibre (37%) as key nutritional attributes. For non-consumers (7%; 34/463), taste, a lack of knowledge of how to prepare and include legumes, and the time taken to prepare, along with family preferences, hindered consumption. Participants identified the food category as “beans” rather than “legumes”, and this may have implications for dietary guidance at an individual and policy level. Addressing barriers to consumption, perhaps through food innovation, emphasizing positive health attributes, and clarification within dietary guidelines, are important considerations for increasing consumption of legumes.

## 1. Introduction

“Legume” is the inclusive term for pulses (the dried non-oil seed of legumes) and all other forms of beans and peas from the Fabaceae (or Leguminosae) botanical family. Legumes have been consumed for at least 10,000 years and are among the most extensively used staple foods worldwide, both for food and animal feed. Consequently, there are thousands of different species and a wide variety of pulses that can be grown globally, making them important for both their economic and nutritional value [1]. A growing body of evidence highlights the nutritional quality of legumes. They provide a rich source of energy, protein, dietary fibre, and slowly digested carbohydrate, with a low glycaemic index [2]. Legumes are a good source of B-vitamins, niacin, folic acid, thiamine, and riboflavin, as well as an array of minerals such as iron, zinc, calcium, magnesium, phosphorous, and copper [3,4].

The health benefits of consumption have been widely documented in the scientific literature. Legumes included in feeding studies have been shown to reduce concentrations of Low-Density Lipoprotein (LDL)-cholesterol [5], blood pressure [6,7], and inflammatory markers [8], have a positive influence on satiety [9], and reduce snacking [10]. In prospective studies [6,11] consumption of legumes has been associated with lower risk of coronary heart disease [12], in particular, when compared with consumption of animal proteins such as red meat [13]. Longitudinal studies of older people from Japan, Sweden, Greece, and Australia concluded that legume intake was the most protective dietary factor for longevity, with every additional 20 g of legumes eaten daily reducing risk of death by 8% [14]. Other trials are supportive of benefits for colon health [15] and in the area of primary prevention for diabetes, body weight management, and cardiovascular disease [16,17]. Furthermore, a 2017 Canadian cost-of-illness analysis study demonstrated that regular consumption of legumes (100 g/day) by 50% of the population, in combination with a low glycemic index or high fibre diet would facilitate savings specifically relating to type 2 diabetes and cardiovascular disease healthcare and loss of productivity costs of over $370 million Canadian dollars [18]. The increased emphasis on legumes suggested in this cost–benefit analysis is apparent in the recently updated 2019 Canada’s Food Guide where messaging around “protein foods” emphasizes plant-based protein foods for their health benefits. The list includes beans and lentils as the first examples of these foods, before nuts and seeds, lean meat and poultry, fish, eggs, and dairy foods [19] as a firm initiative to increase consumption in that country.

Legume consumption has traditionally been higher in food cultures such as Mexican (refried kidney beans), Indian (dhal and pappadums) [20,21], the Mediterranean (navy bean soup), and the Middle East (falafel and hummus) [22], where their use is supported by cooking methods and meals from the local cuisines. A 2019 evaluation of 195 countries found an optimal level of legume intake (defined as 50–70 g daily) in the Caribbean, tropical Latin America, South Asia, western and eastern sub-Saharan Africa [23]. However, in Australia, the per capita consumption sits at 2.9 kg per year compared with a world average of 5 kg per year [24]. Data from the 2011–2012 National Nutrition and Physical Activity Survey (NNPAS), a sub component of the Australian Health Survey (AHS), the largest and most comprehensive health and nutrition survey conducted in Australia, indicated that less than 4% of the population reported consuming the minimum recommended serves of vegetables (which includes legumes), with an average of 2.7 serves of vegetables and legumes/beans from non-discretionary sources [25] compared to the suggested five. Further analysis of this data indicates that Australian adults’ median legume intake was reported as approximately 4 g per day, with 44% of the population sampled reporting they do not consume legumes (Unpublished data). Consumption research of just over 1200 Australians (matched with Australian Bureau of Statistics data) conducted by the Grains & Legumes Nutrition Council (GLNC) found that only 28% consume legumes regularly, revealing a significant proportion of the population as non-consumers [26]. Irrespective of efforts to promote consumption, levels are also low in other countries, with large segments of the population reporting as non-consumers. For example, in Canada, only 13% of the adult population report consumption of pulses (at an average of 113 g) on any given day [27]. In the USA this figure sits at just 7.9% with data from the National Health and Nutrition Examination Survey (NHANES) in 1999–2000 suggesting American adults consumed an average of 0.1–0.3 serves (20–60 g) of legumes each day, one third or less of that recommended [28].

In national dietary guidelines, foods that share common nutritional or biological compositions are often grouped together [29]. Globally, legumes have been included within the meat and meat alternatives group due to their protein content (Bulgaria, Canada, Ireland), in both meat and alternatives, and the vegetable group (Australia, Nordic countries, United Kingdom, United States), and even alongside starchy foods due to their dietary fibre, such as the “cereals, millet, and pulses” food group in India. Finally, Brazil, Spain, South Africa, and Greece place legumes into a separate food group altogether [30]. The decision to include legumes in more than one food group has been justified as a means to promote consumption [31,32] however, this has long been thought ineffective and confusing [4,18,29,31]. Interestingly, in Australia, the serving size guidance also differs for each food group where legumes are allocated. When consumed as a vegetable, the amount is 75 g (cooked weight) aligned with other options within the vegetable group, whereas when legumes are consumed as a meat alternative, the suggested serving size is 150 g [33] to provide significant protein. More recently, an international group have suggested aligning serve size guidance for legume consumption worldwide at 100 g, or half a cup of cooked legumes [30], and promoting them as an independent food group has been considered [18]. When the last Australian Dietary Guidelines were published in 2013, a recommendation to increase consumption by 470% to meet the levels proposed through dietary modelling for the population was indicated [34].

There is a surprising lack of consumer research focused specifically on legumes and legume foods, although this type of research is necessary to understand the barriers and opportunities for promotion to increase consumption. A paper published in 2015 examined the barriers, attitudes, and consumption of lentils in families in Canada [35], and reported 85% of respondents as non-consumers due to lack of knowledge and a belief that family members would not consume them. Cross-over feeding studies using chickpeas [9], and pinto and navy beans with carrots as a control [9,36] were able to draw out more specific issues concerning perceptions regarding both negative and positive aspects of consuming these specific legumes, including the incidence of flatulence, improved gut function, and satiety as drivers for and against. Whereas a focus on particular varieties of legumes may help identify some of the issues regarding consumption, it may also limit the scope of possible responses and other opportunities to assist with increasing consumption. The current study aimed to investigate consumption, knowledge, attitudes, and culinary use of legumes in Australia.

## 2. Materials and Methods

### 2.1. Study Design and Sample

A cross-sectional survey of Australian adults using an online computer-based survey (Appendix A), was used with questions designed to explore relevant themes but also to obtain relevant data to support future marketing campaigns and guidelines for consumption. Consultation prior to survey release with industry stakeholders (including food industry and a pre-competitive group) and piloting by several nutrition professionals, including dietitians, aimed to reduce participant burden and tested face validity. An estimated completion time of 15 min was derived from pilot survey results. A random draw prize incentive ($50 legume basket) was offered to encourage participation. The survey was approved by the University of Wollongong Human Research Ethics committee (project number: 2018/194).

### 2.2. Data Collection

The survey was promoted to adult participants >18 years using three strategies: (i) online via advertisement on social networking platforms Facebook, Instagram, and Twitter and via snowballing; (ii) face-to-face at first year university lectures with a QR code linked with the online survey tool; (iii) via advertisement in the GLNC (not for profit charity) newsletter to ~3000 email subscribers. In all instances, a “click to website” advertisement over a 14-week period from May to July 2018, invited users to complete the survey. Interested participants clicked the advertisement which directed them to the plain language statement and consent form. After providing consent, the participant was directed to the online survey.

Nutrition professionals were excluded from participating to ensure the findings were more reflective of Australian consumers. The survey was comprised of 31 questions, including demographic characteristics including gender, age group, employment status, highest level of education, ethnicity/cultural background, and an indication of the main household purchaser and preparer of ingredients/meals. Questions focused on types of legumes, broad food preferences, legume consumption patterns, nutritional knowledge, attitudes, and culinary use of legumes. Questions in the survey included multiple choice, check multiple box selection, rank and scale, 5-point scale ranging from “strongly agree to strongly disagree”, and free text open answers.

As data collection progressed, online paid advertisements via social media were targeted towards males and participants over 55 years of age to encourage participation from more broad demographic groups.

### 2.3. Data Analysis

Raw data was collated and data cleaning performed using Microsoft^®^ Excel^®^ spreadsheet 2013 (Redmond, WA, USA). Descriptive statistics were used to analyze participant characteristics for frequency counts and percentages. Themes were identified in qualitative responses utilizing data visualization methods, including content or word clouds. Content or word clouds were created to visualize associations in consumer responses surrounding drivers for legume consumption. Content analysis of responses was used to explore topics and themes as they emerged in the data. This represented a conventional approach to content analysis with no preconceived ideas as to the relevant themes derived from responses [37].

## 3. Results

### 3.1. Participants

Of the 505 participants that attempted the survey, 308 answered all questions, leading to a 61% completion rate. While only 197 participants answered most questions, responses were not dependent on each other, and so partially completed surveys were included in the final analysis. Therefore, the numbers of participants who answered individual questions varied, and results are presented as the number of responses to a criterion within the question (for example, male, female, or prefer not to answer) as a percentage of the total number of participants who answered that question, followed by the n values. The majority of respondents were female (78%; 389/499), employed at least part-time (71%; 356/501), and described themselves of Anglo-Saxon heritage (Table 1). Around half of participants also held a Bachelor degree or higher (53%; 264/501), which is significantly above the Australian average of 24% [38]. Mean age of participants was 39 ± 16 years. Three quarters of participants (74%; 340/461) identified themselves as the person who prepared the majority of meals and the individual who purchases the majority of groceries/food within the household (74%; 342/461) (Table 1).

When participants were asked if they follow a specific diet, the majority (74%; 360/486) indicated they followed a “normal diet” (Table 1), whilst one quarter (26%; 126/486) identified following a specific diet, a small proportion (16%; 78/486) of these individuals followed vegetarian or vegan diets. Almost one quarter of respondents (23%; 112/486) acknowledged having dietary restrictions. Participants (n = 102) specified lactose or dairy (26%) and gluten (17%) as the most commonly restricted foods; with a small proportion (1%) identifying dietary restrictions for ethical reasons. When asked about food avoidance, almost half (47%; 226/483) of participants acknowledged avoiding particular foods. The most commonly avoided foods were animal or meat products (such as red meat) (12%; 58/483), dairy products (6%; 30/483) and gluten or wheat containing foods (4%; 19/483).

### 3.2. Legume Consumption Patterns

The vast majority of participants (93%; 429/463) identified that they consumed legumes (“legume consumers”) with only a small proportion identifying as non-consumers (7%; 34/463). Of the legume consumers, almost half (47%; 177/376) consumed legumes 2–4 times each week, almost a quarter (23%; 86/376) consumed legumes more than 5 times per week, whereas 18% (67/376) consumed legumes only once per week. Participants identified the types of legumes they consumed; chickpeas were the main legume participants identified consuming (85%; 360/424), followed by green peas and kidney beans (76%; 320/424) and lentils (74%; 313/424). Over half of these respondents selected baked beans (57%; 241/424) and edamame (54%; 228/424). Pinto and mung beans (17%; 71/424) and adzuki beans (11%; 47/424) were consumed by fewer participants.

### 3.3. Legume Knowledge and Understanding

Participants most commonly associated legumes with foods such as “beans” (72%; 329/455), “lentils” (55%; 252/455), “chickpeas” (42%; 191/455), and “peas” (24%; 109/455). When asked to select the nutritional quality of legumes, consumers chose protein (37%; 169/461) and fibre (35%; 163/461), as well as legumes as a meat alternative (11%; 51/461) and food high in carbohydrate (7%; 31/461). The majority of participants identified “beans” as the term that they would prefer to use when referring to legumes (70%; 321/460), followed by “legumes” (58%; 263/460), “peas” or “pulses” (30%; 140/460) and “lentils” (3%; 12/460).

### 3.4. Consumer Attitudes Related to Legumes

**Figure 1:**
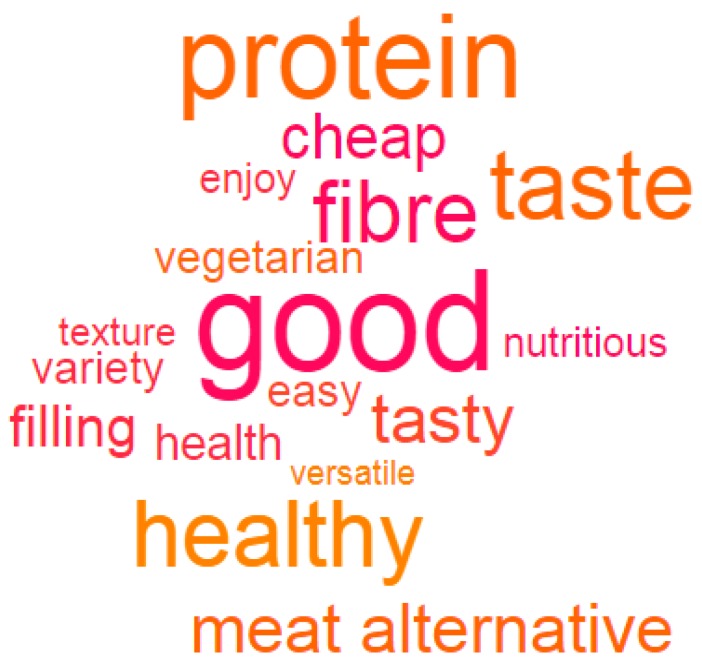
Word cloud based on associations with legumes. Larger font indicates high frequency of mentions by participants. Associations are based on all responses to the question: “We are interested in understanding why Australians eat legumes, please tell us below the main reasons why you eat legumes”. Duplication of one or more words by respondents was taken as an individual instance of association.

A number of themes emerged from the data across multiple questions indicating participants’ knowledge and attitudes to legumes, and the motives for selecting legumes (Table 2). These were identified consistently from a large proportion of responses (n = 401), firstly aligned with health benefits, particularly the notion that legumes are high in protein and/or fibre (Figure 1), and secondly, the ethical reasons for selecting legumes. The use of legumes as a protein source or meat alternative were commonly mentioned. Taste and enjoyment were supported by the notion that legumes were considered filling or added texture and bulk to meals, as well as being affordable. Ease or convenience, both in cooking and preparation, as well as versatility and variety, were also cited.

Positive beliefs and attitudes regarding consumption as well as perceived health benefits of consuming legumes were prevalent in this sample (Table 3). Most participants (98%; 399/409) agreed with the statement that legumes are nutritious and healthy. Similarly, over ninety percent of participants agreed with a statement that legumes are a good source of protein (95%; 387/408) and this was the health benefit participants most strongly associated with legumes. Although over half of participants strongly agreed with the statement “help control your blood sugar”, unprompted, only 4/401 participants referred to glycaemic index or low glycaemic index food as a driver for consumption. Taste was another strong consumption determinant and the majority of participants (89%; 362/409) agreed this influenced their legume consumption.

Participants were asked to rank the most important factors when deciding whether to eat legumes (ranking from 1 most important–6 least important). Health benefits were ranked first by over one third of respondents (34%; 137/407), followed by taste (23%; 92/407). A good source of protein was ranked third (10%; 39/407) and this was followed by convenience and ease of preparation (6%; 23/407).

Non-consumers were asked to identify reasons for non-consumption (*n* = 34) prior to exiting the survey. Taste was identified as a barrier for some participants (41%; 14/34) (Table 2). However, a lack of knowledge and skills, such as not knowing how to prepare them and the time (both perceived and actual) it takes to prepare legumes, or how to include them in the diet, was also cited by participants (35%; 12/34). The influence of others, such as family preferences, also played a role in not choosing to consume legumes (15%; 5/34).

### 3.5. Legume Culinary Use

When asked what time of day legumes were consumed, the majority of participants consumed legumes at dinner (95%; 352/372), followed by lunch (69%; 257/352), and then breakfast (40%; 150/372). Participants then specified which legumes were consumed at different meal times, baked beans were most commonly consumed at breakfast (75%; 113/150). Chickpeas were most commonly consumed at lunch (37%; 95/257), and lentils were the main legume used in the dinner meal (43%; 151/352). This data is in alignment with the most highly consumed legumes identified by consumers in the survey. Participants identified the types of dishes they prepare with legumes, most selected soups (75%; 271/362), salads, and curries or stews (71%; 258/362), followed by side dishes (66%; 239/362) or as a main dish (63%; 229/362).

When participants responded to statements regarding preparing and cooking legumes, most agreed with the statements, “I know how to prepare or cook legumes” and “I find legumes easy to prepare/cook” (76%; 286/377). Over half of participants agreed with a statement about preferring to use canned legumes (58%; 218/377) whilst approximately half of the participants disagreed with the statement “it takes too long to cook legumes” (55%; 207/377).

Participants identified cultures or cuisines that might influence their legume consumption. Mexican culture and cuisine was identified by almost half of respondents (47%; 157/332), followed by Indian (34%; 112/332) and Middle Eastern (10%; 34/332).

### 3.6. Purchasing Behavior and Sources of Information

When asked where legumes were purchased for home use, most participants (95%; 360/377) purchased legumes from supermarkets, or from a specialty (17%; 58/377) or an ethnic/cultural grocery store (14%; 48/377). These were purchased in canned (91%; 342/377), dried (62%; 234/377), frozen (41%; 154/377), or fresh (35%; 130/377) form. When asked where information and recipes on legumes was obtained from, internet websites (68%; 255/377), family or friends (65%; 246/377), personal knowledge or experience (59%; 221/377), and cookbooks (50%; 188/377) were cited.

## 4. Discussion

This study provides insights from the largest online survey undertaken in Australia to date specifically exploring consumption, knowledge, understanding, attitudes, and culinary use of legumes. Consumer beliefs about plant foods including legumes have been previously explored through focus groups, with similar findings concerning lack of knowledge of legumes, particularly how to prepare legumes and issues concerning the extended time needed in preparation [39]. The current study extends those findings focusing specifically on legumes. The sample of Australian consumers surveyed perceived greater benefits of legume consumption than barriers, with many incorporating legumes into their diet. Overall, consumer attitudes towards legumes were positive, particularly in relation to their perceived health attributes, as well as ethical or environmental motives, taste, enjoyment, and versatility. However, it is worth noting that this sample was not representative of the Australian population and thus the frequent consumption of participants overstates the reported consumption from NNPAS 2011–2012 (4 g/day) [25]. Instead, these results provide an extrapolation of regularity of consumption, rather than a precise value of intake. The study provides valuable insight into preferences for legume consumption, knowledge and understanding, attitudes, culinary use, and reasons for non-consumption from a small group of participants (7%; 34/463). They provide promising insights into why these respondents do consume legumes and this may provide leverage for encouraging consumption in other groups.

The inclusion of legumes as part of both the vegetable and meat alternatives group is supported through the Australian Dietary Guidelines, with evidence for legumes within the diet for overall health [33]. However, there are suggestions that individualizing advice for vegetables, in particular for legumes, may improve consumer understanding and consumption [4,29,30]. As consumption of legumes may assist in managing weight and reducing the risk of obesity, an independent risk factor for non-communicable diseases [40,41], there is significant impetus to encourage consumption from this food group. In fact, the growing body of evidence within the Global Burden of Disease data suggests that the consumption of vegetables including legumes is important to improve health outcomes in the Australian population [42] and globally [23].

Dietary guidance aside, the most important factors influencing food choice have been identified in the literature and include taste, health benefits, and nutrition, as well as time and convenience [43,44], and these are also reflected here regarding legumes. Interestingly, despite low glycaemic index as a significant nutritional benefit [45,46,47], unprompted, few participants identified this feature, and many participants responded with neutral agreement to a statement surrounding the health benefits of legumes for blood sugar control. This may indicate that either glycaemic index is not well understood, is not a strong motivator for legume consumption, or it is an attribute not typically associated with legumes. Conversely, cost and affordability was a recurrent theme in participant responses as a secondary driver for legume purchase and consumption in this study, despite this aspect being considered a negative attribute in studies from other countries and within lower socio-economic groups [48]. Therefore, while this aspect alone may not be the most compelling reason for purchase, in Australia the relative low cost is likely important.

The barriers to consumption, including lack of knowledge regarding preparation of legumes and the perceived time associated with cooking, are both opportunities for education and indicate consumers need support in selecting and consuming legumes (both from dried and canned form). These findings were similar to literature examining vegetable consumption in a qualitative study exploring plant food intake [39]. The study explored Australian consumers’ perceived barriers and benefits of plant foods (including vegetables, grains, legumes, nuts, and seeds) and found participants viewed legumes as difficult to incorporate into the diet due to taste barriers, the perceived lengthy preparation required and lack of knowledge of how to prepare them to be palatable [39]. Similarly, Canadian research explored factors influencing pulse consumption through online interviews and focus groups, and found that taste and general dislike was the number one barrier for non-consumers, followed by not knowing how to prepare or cook them [49].

Gastrointestinal side effects such as flatulence was not a major issue in this study, but it has been raised in other studies, although it has also been acknowledged that symptoms resolved with time and this needs to be emphasized by health care professionals when providing advice regarding legumes [10,50]. Polak et al. offered practical suggestions for increasing pulse consumption, such as keeping dried legumes in the pantry, particularly lentils as they cook quickly; adding canned legumes to salads, pasta or stews; and suggests saving time by soaking and cooking more lentils than necessary and freezing the leftovers in portions to be used later [51]. It is recommended that nutrition professionals gain experience in cooking with legumes and in addition to promoting their nutritional value, and are able to discuss multiple recipe ideas for specific types of legumes with their clientele [50]. Here, we also reviewed the type of legume most often consumed with meals, for example baked beans at breakfast, chickpeas at lunch, and lentils with dinner, which points to a further opportunity for promotion of specific legume types with specific meals, which may be popular with consumers.

Ironically, more than ten years ago researchers proposed the strategy of making plant foods trendier to increase vegetable and legume consumption [39]. Two emerging key global trends in nutrition and health included “plant based” and “plant protein”, and these have been gaining traction as drivers for purchasing decisions among consumers who are starting to embrace legumes in their diet more frequently due to their known “nutritional halo” including dietary fibre, vitamins, and minerals [52]. Recent research identified 300 new products launched in Australia between 2012 and 2017 that contained either vegetables and legumes, or legumes on their own, where at least half a serve (1 serve = 75 g) of legumes featured as the main ingredient [24]. Legumes have been incorporated into new product formulations, leveraging the plant protein trend, with the emergence of legume based pasta products, breads, and legume snacks, illustrating food industry’s willingness to incorporate legumes to meet consumer needs and demands [52]. These findings were reflected in a recent supermarket audit of all products on shelf in the legumes category, which identified 299 legume food products, a 49% increase in numbers of products since the previous audit in 2017. The increase was particularly evident in the snacks category (including dips, chips, and whole roasted legumes), which grew from 75 products in 2017, to 128 in 2019, a 66% increase in product numbers [53]. As with the findings in this study, others have noted that legumes, such as chickpeas, are becoming more common (or are more commonly consumed) via products such as hummus dip [24]. Consumers may more readily identify with specific legume types that are used in single recipes or cuisines. This suggests that in dietary assessment, very specific questions may need to be utilized, rather than asking more generally about legumes as a whole food group. Also, some consumers may in fact be consuming legumes in crushed or powdered form within various food items without realizing, such as in legume flours used in bread, breakfast cereal products, legume based pasta products, or legume snacks now available on the market [24]. While the inclusion of legumes as an ingredient within foods may be the most effective option to increase consumption in the longer term, this may have important consequences and add complexity to tracking changes in consumption of legumes in future National Nutrition Surveys [10].

A proportion of participants in this sample identified as vegetarian or vegan (16%; 78/486), and themes concerning ethical and environmental influences emerged throughout participant responses. This is consistent with population trends, which indicate that vegetarianism and veganism is on the rise [54]. Market research conducted in 2016 found that the number of Australian adults with vegetarian or almost vegetarian diets increased from 1.7 million to 2.1 million (11.2% of the total population) from 2012 to 2016 [55], and this number is anticipated to increase in alignment with global research findings. The trend towards eating less meat or a “flexitarian” diet approach is also being adopted by more Australians and legumes are well placed to provide dietary variety and enable nutritional adequacy, not only for individuals that adopt these lifestyle choices, but for all Australians to improve the nutrient density of a healthy diet.

Verain et al. noted that sustainability of food systems is an increasingly important issue. Population food consumption is considered an important factor in shaping the sustainability of our food supply [56], as the environmental, social and economic consequences of food production and consumption are starting to demand attention. The adoption of sustainable dietary patterns such as meat curtailment or adopting a more plant-based diet has gained traction in recent years, and participants acknowledged the role that legumes can play as a meat alternative and dietary protein source [57]. As Marinangeli et al. highlights, legumes could be utilized as a nutrient-dense food source that, in combination with other efforts, could help contribute to more sustainable dietary patterns in the future [30].

### Strengths and Limitations

Online electronic surveys are a useful tool in research as they have the ability to reach a larger number of people and allow for wide geographic coverage, enabling data to be collected quickly and efficiently from respondents. Social media as a method of dissemination also allows for demographic targeting as was used in this study to obtain responses from harder to reach age groups. However, as acknowledged, there are some key limitations. The consumers who were drawn to participate in the survey were more likely established legume consumers and may have been generally more interested or motivated in areas of nutrition. This was most obvious in the sample bias, including a high proportion of legume consumers as opposed to non-consumers, and not in alignment with national consumption data. It was also apparent that a younger demographic, a greater proportion of female respondents, and a high proportion of tertiary educated individuals responded to the survey. Over half the respondents were aged between 18–35 years and held tertiary qualifications. In contrast, a limited number of participants over 65 years responded indicating that future research should target older Australians. The overall length and demand of the survey may have also contributed to the 75% completion rate.

## 5. Conclusions

This study aimed to investigate consumption, knowledge, understanding, attitudes, and culinary use of legumes in Australia. Findings suggest consumers perceived many health benefits linked with legume consumption and were able to correctly identify key nutrients in legume food choices. In order to improve and encourage intake, food industry, public health advocates, dietetic professionals, and nutrition educators should focus on these health benefits and potentially environmental benefits, as well as correcting the perceived barriers to consumption. Barriers including lack of preparation and cooking knowledge, as well as time and convenience, have a strong influence on food choice but are modifiable. Shifting consumer behavior and improving familiarity with legumes will require a range of initiatives from cooking demonstrations, to recipe ideas as well as educational advice. Food industry manufacturers should also continue to engage in providing a variety of legume food choices, with particular emphasis on convenience-focused products but also incorporating legumes into core foods aligned with Australian Dietary Guidelines. Public health policy and other strategies are required to improve familiarity and to encourage consumption of legumes, perhaps starting with more clear dietary guidance at a national level helping to highlight the unique nutritional profile of legumes within the overall dietary pattern. Thus, it may be timely for legumes to be separated from other food groups with specific and clear recommendations for consumption if not daily, then weekly, within food guidelines.

## Figures and Tables

**Table 1:** Demographic characteristics of participants (*n* = number of participants responding to each specific question).

Demographic Variable	Frequency * (%) **
Gender (*n* = 499)	
Male	109 (22)
Female	389 (78)
Prefer not to answer	1 (0)
Age in years (*n* = 499)	
18–25	136 (27)
26–35	122 (25)
36–45	90 (18)
46–55	77 (15)
56–65	46 (9)
65 or older	28 (6)
Employment Status (*n* = 501)	
Employed, working full-time	219 (44)
Employed, working part time	137 (27)
Not employed	31 (6)
Retired	34 (7)
Student	73 (15)
Carer or not able to work	4 (0.8)
Prefer not to answer	3 (0.6)
Education Status (*n* = 501)	
Some high school	15 (3)
Completed high school	96 (19)
Certificate or diploma	126 (25)
Bachelor degree	183 (37)
Master’s degree/PhD	81 (16)
Cultural Background (*n* = 426)	
Anglo-Saxon/Caucasian(Australia, NZ, UK, US/Canada)	277 (65)
Portuguese	15 (4)
Italian	12 (3)
Greek	10 (2)
Other European Decent	69 (16)
India/Sri Lanka	8 (2)
Other Asian Decent	18 (4)
Other	17 (4)
Diet (*n* = 486)	
No Specific Diet	360 (74)
Vegetarian	41 (8)
Vegan	37 (8)
Other	20 (4)
Gluten Free	17 (4)
Pesco-Vegetarian	7 (1)
Low FODMAP	4 (1)
Household demographics (*n* = 461)	
Main household cook	340 (74)
Main household food purchaser	342 (74)

* Frequency of responses to each criterion. ** Percentage (%) of responses to question.

**Table 2:** Consistent themes emerging from the data and keywords that summarize participant responses related to legume consumption, knowledge, and attitudes.

Theme	Subthemes/Keywords	Exemplar Quotes
**Health Benefits**	Health or Healthy Protein Fibre Vitamins Nutrients or Nutrition Good for you	*“I eat legumes because it promotes a healthy balanced diet. They also are healthy”* *“They are healthy. They are high in fibre”* *“I enjoy them and the variety they add to my diet plus the health benefits—high fibre and protein and nutrient rich”* *“As a source of protein, fibre and vitamins since I’m a vegetarian”* *“They taste good, add flavor/texture to the meal they are in, extra nutrients/they’re good for you”* *“Because they provide a great range of nutrients that are good for my body. Being pescatarian, I am also wary of protein and iron too, so legumes are a good choice. Love that they are fibre-rich so they fill me up”*
**Ethical/Environmental**	Meat alternative Meat replacement Vegetarian/ Vegan Sustainable Environmental	*“I mainly eat vegetarian at home and I choose beans and peas as a useful plant protein source”* *“They are high in protein and because I’m vegan, this is a healthy alternative to meat”* *“They are very filling and an important substitute for meat in my diet”* *“I think they are healthy and filling that that eating them will help me in my effort to eat less meat, and it is better for the environment overall as their production requires less energy and water compared to meat products”*
**Enjoyment/Taste**	Taste Filling Enjoyment Texture	*“They taste good and are good for you”* *“Taste, texture, health benefits”* *“Taste, how it adds different texture to dishes, nutritional value, meat alternative, supporting local industry”* *“I like them, they are filling and nutritional. Also, they are great at absorbing flavor, so excellent for increasing size of a meal to feed more people without having to add extra meat”* *“They are essential for good health and I enjoy eating them”*
**Cost**	Affordable Inexpensive	*“Tasty, cheap, source of protein”* *“Convenient, easy and cheap source of protein”* *“Canned is extremely convenient and very affordable (<$1 per can)”* *“Relatively cheap to buy in the tin, good to add to soups or curries to fill it out”*
**Ease/Convenience**	Convenient Easy to prepare or cook	*“Healthy, cheap and relatively easy to prepare”* *“I use canned products because they are easy and convenient”* *“They are easy to prepare, just time consuming, hence the convenience of using it from the can”*
**Versatility/Variety**	Variety Versatile Flexible	*“Vegetarian, cheap source of good protein, very versatile (can use in pasta, soups, on toast etc.)”* *“They’re tasty, versatile, and they’re an easy way to add extra nutrients to my meals”* *“They’re very flexible and can be used in many different styles of cooking”*
**Culture/Tradition**	Cultural Cuisines (Mexican, Indian, Middle Eastern)	*“I love foods from many different cultures and enjoy legumes in too many ways to list.* E.g. *Indian, Mexican, Middle Eastern”* *“I’m Spanish and we regularly cook a traditional dish with green lentils. I do enjoy Mexican and Indian dishes”* *“I grew up never trying many other cultures foods. Now I cook a lot of Indian food which involves lentils, I also use legumes to substitute meat”*
**Barriers to consumption**	Taste and/or texture Lack of knowledge or skills in preparation or cooking Time consuming in preparation and/or cooking Influence of others e.g., family preferences Intolerance or GI symptoms High in carbohydrate	*“Don’t fit my life at the moment”* *“Kids not that keen on them.”* *“Using dried legumes is tedious, confusing and never seems to work”* *“I don’t know much about cooking legumes”* *“The time to soak dried legumes is a limiting factor for me”* *“Wish I knew more creative and tasty ways to use them more”* *“Mainly buy pre-made—I don’t like to cook them”* *“I hate cooking legumes and usually buy things premade”* *“My partner has intolerance issues”* *“High carbohydrate. Only eat rarely”*

**Table 3:** Level of agreement with beliefs and attitude statements related to consumption and health benefits of consuming legumes *.

**Question:**	**Agreement (%)**		
**How Much Do You Agree with the Following Statements about Why You Choose to Eat Legumes**	**Strongly Agree or Agree**	**Neutral**	**Strongly Disagree or Disagree**
Legumes are nutritious and healthy	97.6	2.2	0.2
Legumes are a cheap and affordable food	89.3	9.3	1.4
I like the taste of legumes	88.5	10.0	1.8
My family likes eating legumes	64.3	23.2	12.5
I am trying to choose vegetarian meals more often	54.3	23.2	22.5
Legumes are part of my traditional diet or I grew up eating them	43.4	20.1	36.5
**Question:**	**Agreement (%)**		
**How Much Do You Agree with the Following Statements about the Health Benefits of Eating Legumes**	**Strongly Agree or Agree**	**Neutral**	**Disagree or Strongly Disagree**
Give you a good source of protein	94.9	4.9	0.2
Be a good vegetarian option/meat alternative	86.9	10.4	2.7
Help you feel full	88.2	10.6	1.2
Increase bowel movements	79.6	19.7	0.7
Benefit your gut/digestion	76.5	21.8	1.7
Help control your blood sugar	52.7	46.3	1.0
Help lower bad cholesterol	51.2	47.5	1.3

* Data presented as a % of total number of responses (*n* = 409).

## Appendix A

Survey Questions

Q1. How did you hear about this survey?

Q2. What is your age group?

Q3. What is your gender?

Q4. Which of the following categories best describes your employment status?

Q5. What is the highest level of education you have completed?

Q6. Do you follow a specific diet? (For example, vegetarian, vegan, special diet for a medical condition etc.)

Q7. Do you have any dietary restrictions? Please tick what options apply:

Q8. Do you avoid any particular foods?

Q9. We are interested in how different cultures use and eat legumes. We all live in Australia, but can come from many different cultural backgrounds. What is your main cultural background? If you prefer not to answer, please leave blank.

Q10. When thinking of legume foods, what foods come to mind? Please list below

Q11. Legumes have a number of nutritious qualities. When you think of legumes as a food, how do you think of them? Please tick the statement you associate the most with legumes, “Legumes are...”

Q12. Legumes can be known by a few different names, how do you refer to legumes or what do you call them? Tick which options apply.

Q13. Are you the main household cook?

Q14. Are you the person in your household who purchases the majority of the groceries/food?

Q15. This question will ask you about finding legumes when you are grocery/food shopping in the supermarket/store. How much do you agree with the following statements?

Q16. Do you sometimes or regularly eat legumes? (These include foods such as hummus, baked beans, pea soup, falafels, dhal, refried beans and tofu pictured above)

Q17. If yes, what types of legumes do you eat/enjoy? Please tick legumes you eat from the list below. Images have been provided to help to identify them.

Q18. If no, what are the main reasons you don’t eat legumes (beans, peas, lentils and chickpeas)? Tick all options that apply.

Q19. We are interested in understanding why Australians eat legumes, please tell us below the main reasons why you eat legumes

Q20. How much do you agree with the following statements about why you choose to eat legumes?

Q21. Which of the following factors is most important to you when deciding whether to eat legumes? Please Rank 1 - 6 from most important to least important.

Q22. How much do you agree with the following statements about the health benefits of eating legumes, “Eating legumes can…”

Q23. On average, how often do you eat legumes at home? If you are not sure, please provide your best estimate

Q24. What time of the day do you eat your legumes? Please answer all that apply: (For example, you may eat baked beans at breakfast or chickpeas with lunch)

Q25. Where do you usually purchase legumes you use at home?

Q26. We are interested in understanding what form you normally buy legumes in, please tick which options apply.

Q27. What types of dishes do you make with legumes? Please answer what options apply and list which type of legumes you use to make them. (For example, soups with kidney beans or curries with lentils)

Q28. Can you give some other examples of meals you might prepare or consume including legumes not mentioned previously? (For example, pea and ham soup, baked beans on toast, lentil dhal)

Q29. How much do you agree with the following statements about preparing and cooking legumes?

Q30. What cultures or cuisines might influence your legume eating habits? (For example, “I come from an Indian Heritage where we use legumes commonly” or “I like Mexican dishes and use beans in the recipes”)

Q31. This is the last question, where do you typically get recipes and information about preparing and cooking legumes?
